# A Semi-Self-Supervised Intrusion Detection System for Multilevel Industrial Cyber Protection

**DOI:** 10.1155/2022/4043309

**Published:** 2022-09-21

**Authors:** Fuchuan Ye, Weiqiong Zhao

**Affiliations:** ^1^Information and Educational Technology Center, Southwest Minzu University, Chengdu 610041, China; ^2^School of Intelligent Technology, Geely University of China, Chengdu 641423, China

## Abstract

Industry 4.0 affects all components of the modern industry value chain. The accelerating use of the Internet and the convergence of industrial and operational networks constantly increase the need for secure industrial communication solutions. Therefore, “multilevel industrial cyber protection” is critical to Industry 4.0. In general, industrial protection refers to safeguarding information and data and the intellectual property rights of production processes related to the overall industry environment. The availability, integrity, and confidentiality of systems must be maintained. The goal challenge is the best possible protection from attacks and threats which create immediate financial damage and other risks in the industry (reputation, etc.). Based on the Defense-in-Depth strategy, a holistic, multilayered, and in-depth protection of industrial systems is developed in this paper. Specifically, a Semi-Self-Supervised Intrusion Detection System (S3IDS) is proposed, which combines advanced machine learning techniques for industrial data noise reduction to automate the discovery and separation of classes, which are essentially equivalent to cyber-related anomalies. As demonstrated by a mathematical simulation based on computational number theory and specifically on the concept of the single object, the proposed S3IDS learns to accurately reconstruct samples to predict the nature of an anomaly created directly by the industrial ecosystem.

## 1. Introduction

Historically, industrial companies worldwide have approached cybersecurity in their Information Technology (IT) and Operational Technology (OT) networks very differently [1]. Most companies have already implemented technological infrastructures for detecting and dealing with network threats, but, for their industrial (OT) systems, coping with cyber threats is usually limited to isolating the relevant procedures from the rest of the network. Industries are constantly being “digitized” by investing more and more in intelligent technologies, new automation systems, and other applications that promote productivity growth or improve many other indicators of interest to the organization [2]. This rapidly equates IT systems with OT systems, making the latter more vulnerable to attacks that formerly solely affected the former [3].

Cyberattacks on industrial organizations are considered a perilous threat [4], as they have the potential to cause significant material losses and lead to disruption of the production cycle of the entire system [5]. They target, among others, industrial control and data collection systems (ICSs, SCADA) [6, 7]. In addition, due to the sensitive information available to industrial organizations, they are usually an attractive target for attackers [3].

The situation to date focuses on the human aspect, experience, and expert opinion, using assistive technology to analyze and reduce risks and dangers to industrial infrastructure [8, 9]. For optimal results with this methodology, there should be up-to-date threat intelligence, incident reports, and vulnerability warnings, which will feed indefinitely the power grid monitoring tools and in-depth human oversight and intervention from cybersecurity staff [10].

The above passive function, in combination with the new class of requirements in cybersecurity, leads to the logic of adopting solutions that include fully automated security methods based on advanced techniques of artificial intelligence [11], with the parallel minimization of human intervention [12]. The idea of getting rid of the constant surveillance and direct presence of people is related to advanced attacks like Stuxnet and BlackEnergy, where it turned out that it just needed an infected USB stick or open a phishing e-mail to allow the attacker to access an isolated industrial network [5]. In addition, throughout the last several months, we have witnessed, in many cases, highly specialized attacks on systems and infrastructures that use industrial protocols [4, 13].

A great example is the largest colonial gas pipeline in the USA, which was shut down for several days after a malicious cyberattack and attributed to the shadow criminal group DarkSide [14–16]. Also, in the first quarter of 2021, in the city of Oldsmar in Florida, there was an attack on the government infrastructure responsible for the city's water supply. In essence, a remote attempt was made to change the mix of related chemicals with water disinfection, resulting in the mass poisoning of consumers [7, 15, 17, 18].

Just months ago, another cyberattack occurred in a public hospital in Israel. The specific attack created significant problems in the smooth provision of services of the organization, while it required the payment of a certain amount of money (ransomware). Hospital services reacted by using alternate resources and support systems. Fortunately, there was no loss of life in this case, as unfortunately happened at a similar point in a hospital in Germany a few months ago [4, 6, 15].

To deal with these offensive techniques, the research community has proposed various solutions in which machine learning systems operate with self-adaptation procedures and rearrange their mode of operation, depending on the algorithms' hyperparameters that most often specify their mode of operation.

## 2. Literature Review

Several researches have presented adaptive cyberattack detection algorithms to fulfill the requirement for continuing learning paradigm changes [19–21]. Still, they have failed to establish a more comprehensive system of knowledge for detection performance and their evaluation practice [22–24].

In their review of earlier work for threat detection techniques in industrial control systems, Kaouk et al. [3] underlined the difficulties and advantages of putting such solutions into practice. Such information, in our opinion, will be helpful for future studies in manufacturing security. ICS intrusion detection technology is evolving swiftly, but there is still room for improvement. The integration of IDS with ICS will face a variety of risks. Most methods used in the literature are anomaly based, meaning that they look for any notable departure from the norm. To enhance how IDS can react to alarms, techniques that can tell the difference between a flaw and a threat are desperately needed. Another difficulty is that the vast majority of existing IDS are network based and cannot access encrypted data because of this. For instance, encryption use is hampered by hardware limitations. However, new parts of the ICS have begun to offer encryption because of advancements in hardware computation capabilities. IDS must therefore rely on data sources other than Internet activity. The operation of IDS should also be taken into account as ICS grow in size and complexity and comprise geographically dispersed systems. Alternative technologies decentralized and collaborative IDS must therefore be created. Such information, in our opinion, will help advance future studies on the integrity of ICS.

Hu et al. [25] went into more detail on ICS's attributes and security needs in 2018. They proposed a taxonomy of IDS for industrial control systems based on three techniques: protocol analysis, traffic mining, and control process analysis. They also examined the benefits and drawbacks of various IDS categories. They concluded that, despite the rapid advancement of ICS technology, there is still much opportunity for ICS IDS development. It was crucial to construct dispersed and collaborative IDS due to the scattered structure of ICS subsystems. Evaluating associations between distributed IDS, fusing a group of dispersed and potentially contradictory detection findings, and obtaining accurate and real-time complete detection results are a novel and intriguing subject. How to react to warnings is a major problem for ICS IDS. In specific control systems, simply notifying administrators of the alarm may be considered sufficient; nevertheless, automatic reaction mechanisms must be taken into account to ensure the protection and reliability of ICS. How to automatically improve intrusion detection algorithms while they are being used is a crucial topic. To maintain a satisfactory detection accuracy, intrusion detection algorithms must automatically optimize their judgments of changing contexts. ICSs typically need to operate continuously, and the system parameters (such as durable components, access controls, and system constraints) of an objective ICS may change over time. These days, ICSs are internet-accessible, and ICS security concerns are increasingly becoming more critical. Traditional IDS created for IT platforms cannot function well on ICS because of its uniqueness. It can assist ICS in identifying various intrusions and lowering the frequency of industrial mishaps caused by malicious attacks.

Adversarial Machine Learning, often known as cyberattacks over neural network models on Engineering IDS, was examined by Anthi et al. [4]. By constructing adversarial samples and evaluating classification patterns, they studied how adversarial learning may be used to target supervised models. As adversaries could be able to get beyond the defenses, such attacks could have dire effects on ICS systems. This can result in delayed assault detection, which might harm the infrastructure, cause financial loss, or even result in fatalities. An actual electric grid data set was utilized for training and evaluating commonly used unsupervised feature learning classifiers in support of the studies described here. The investigation also studies how adversarial training on such sets can enhance the resilience of supervised models. Using the testing data, adversarial samples were created with various combinations that changed the model's interference and complexity.

According to Ayodeji et al. [4], in 2020, the failure to recognize and distinguish between the intrinsically identical signatures that define normal transients typical of complex systems contributes significantly to false alarms in ICS systems. The majority of machine learning-based detection techniques created for Scada Systems (ICSs) are taught on network packet logs and solely rely on network layer traffic monitoring to identify intrusions. They looked at the most current developments in malware detection algorithms, their shortcomings, difficulties, and the state of their use in crucial infrastructures. Additionally, they started a conversation about the parallels and differences between the growth of computational skills and equipment for classiଁcation and hacking in defense of complex systems and the requirement to distinguish between them clearly. They used nuclear energy controllers as a case study to demonstrate the challenges to a smooth changeover of security algorithms. To significantly reduce the number of annoyance warnings generated, they suggested a method that considers the subtleties in the data utilized in creating machine learning algorithms. The current findings and recommended course of action lay the groundwork for creating robust intrusion detection systems that significantly reduce the problem of false alarms that plague existing intrusion detection systems.

The transition of the ICS from isolated systems to virtualized platforms was closely examined by Bhamare et al. [1], who also noted the considerable efforts made by both business and technology to construct secure ICSs and the relevance of machine learning approaches for ICS cybersecurity. ICS security remains a concern despite the recent popularity of big data insights and cloud computing. Cloud platforms will eventually help ICSs and industries. Still, inadequate security in cutting-edge multicloud platforms could result in expensive security breaches in real-time industry platforms. It is incredibly challenging to prevent and identify assaults at the ICS component level due to the sophistication of emerging viruses attacking control systems, including rootkits and zero-day attacks. New intrusion detection strategies for ICS devices at the production control level are thus required. Additionally, they said that a testbed might help with the difficulties of safeguarding an industrial process by offering more information about how the method is managed with the aid of sensors and control laws and comprehension of the security needs, mainly to handle control using cloud-based services.

An examination of the development and usefulness of security mechanisms that have been put out in both industry and academia was presented by Rubio et al. in [2]. In the past several years, there has been a tremendous advancement in the design of security methods for industrial environments [1]. Advanced solutions like honeypot systems and data correlation systems are integrated into commercially accessible products, but innovative detection techniques and architectures are also created in academia [19]. Research is still needed in several areas, including the viability and incorporation of proactive defenses, the deployment of defensive mechanisms in the IIoT and cloud computing, and the emergence of Industry 4.0[26]. Furthermore, to validate defense mechanisms against Advanced Persistent Threats (APTs) and make them more integrable and usable so they can be readily integrated into more crucial infrastructures, it is vital to take into account existing APTs and APT phases [15, 16, 21].

In this spirit, an approach is needed that with minimal configuration and the necessary training samples each time will be able to create a generalized framework for detecting known and unknown attacks on a network. Based on the above challenge and the Defense-in-Depth strategy, in general, S3IDS is proposed that should be applied in the industry. Using advanced machine learning methods automates recognizing anomalies related to cyberattacks [27]. To prove the applicability, we used mathematical simulation based on computational number theory. Mathematical simulation is a process to identify and predict the behavior, performance, and optimization of some physical or abstract systems corresponding to various scientific and engineering applications.

## 3. Proposed S3IDS

Given the general issues of machine learning systems to deal against serious cyberattacks effectively and with minimal human intervention, this work proposes the creation of an innovative computer intelligence system [28, 29], with minimal human intervention [30–32], significantly strengthening the security mechanisms of network infrastructure [12, 23, 33]. In particular, S3IDS is proposed, an advanced cyber threat detection system, which is a highly innovative tool for operational security. Specifically, we implement a semi-self-adapted machine learning methodology [9–11] based on Semi-Self-Supervised Learning, which may determine the sort of attack based on generic reshaping characteristics generated directly from the unknown online environment and web data [22, 34, 35].

The proposed system's major innovation is based on computational number theory, notably the idea of a monoid object in a category. Monoids are semigroups that have an identity. A monoid is a set containing an associative binary operation and an identity member in abstract algebra, a field of mathematics. For example, nonnegative integers with addition form a monoid, with 0 as the identity member. Such algebraic structures may be found in many disciplines of mathematics. In terms of function composition, the functions from a set create a monoid. In general, the morphisms of an item form a monoid in category theory; conversely, a monoid may be considered a category containing a single entity.

Many abstract data types in computer science may have a monoid structure. A succession of monoid components is “folded” or “stacked” to generate a final value in a recognizable pattern. Many iterative algorithms, for example, must update some “current set” at each iteration. A monoid function may be used to represent this pattern cleanly. In particular, the proposed methodology ensures that the correlation of monoid operations can be predicted using a correlation algorithm, effectively using multiple cores [36, 37].

In particular, if A is a nonempty set, the operation on A for any representation of the form *f*  : *A* × *A* − ⟶ *A*; e.g., addition and multiplication are operations on Z. The value of *f* in the pair (*a*, *b*) will be denoted by *afb*. A pair (*G*, *∗*), where *G* is a set and  *∗*  is one operation on *G*, is called a monoid if the following properties are valid [36, 38, 39]:

(1) $$\begin{array}{c} x*\left(y*z\right)=\left(x*y\right)*z\text{,} \end{array}$$

such as

(2) $$\begin{array}{c} x*e=x=e*x\text{.} \end{array}$$

If there is another element *k* ∈ *G*with the above property, then for every *x* ∈ *G* we have

(3) $$\begin{array}{c} k*x=x=x*k\text{.} \end{array}$$

Thus, we get *k* = *e* *∗* *k* and *e* *∗* *k* = *e*, from where *e* = *k*. Therefore, the element *e* is unique and is called the neutral element of G. If *x* *∗* *y* = *y* *∗* *x* is also valid for every *x*, *y* ∈ *G*, then the monoid (*G*, *∗*)is permutable. So, the pairs (*N*, +), (*Z*, +), (*Q*, +) are substitutively monosyllabic with neutral element 0 and the pairs (*N*, *·*), (*Z*, *·*), (*Q*, *·*) are substitutive monoid.

Respectively, if (*G*_*i*_, *∗*_*i*_) is a monoid with neutral element *e*_*i*_(*i*=1,…, *k*), the set *G*_1_ × ⋯×*G*_*k*_ is a monoid with the operation [40, 41]:

(4) $$\begin{array}{c} \left(x_{1},\ldots ,x_{k}\right)*\left(y_{1},\ldots ,y_{k}\right)=\left(x_{1}{*}_{1}y_{1},\ldots ,x_{k}{*}_{k}y_{k}\right)\text{.} \end{array}$$

Its neutral component is

(5) $$\begin{array}{c} \left(e_{1},\ldots ,e_{k}\right)\text{.} \end{array}$$

If we have a function with the field of definition, the set of positive integers, and a field of values, the set of complex numbers (numerical function), then we denote by A the set of numerical functions, while the numerical function calculates the exponential product of *f* and *g* [36, 37, 39]:

(6) $$\begin{array}{c} f*g:N\setminus \left\{0\right\}⟶C,n⟼\left(f*g\right)\left(n\right)=\underset{ab=n}{\sum }\;f\left(a\right)g\left(b\right)\text{,} \end{array}$$

where the pairs (*a*, *b*) run through all the natural whose product is equal to *n*. The correspondence (*f*, *g*) − ⟶ *f∗g* defines an operation on A, which is called associative multiplication since the pair (*A*, *∗*) is a permutable monoid. If *g*, *h* ∈ *A*, then for every natural *n*  >  0 we have [36, 37, 39]

(7) $$\begin{array}{c} \left[f*\left(g*h\right)\right]\left(n\right)=\underset{ab=n}{\sum }\;f\left(a\right)\left(g*h\right)\left(b\right)\\ =\underset{ab=n}{\sum }\;f\left(a\right)\;\underset{c\;d=b}{\sum }\;g\left(c\right)h\left(d\right)\\ =\underset{ac\;d=n}{\sum }\;f\left(a\right)g\left(c\right)h\left(d\right)\text{.} \end{array}$$

Similarly, we get

(8) $$\begin{array}{c} \left[\left(f*g\right)*h\right]\left(n\right)=\underset{ac\;d=n}{\sum }\;f\left(a\right)g\left(c\right)h\left(d\right)\text{.} \end{array}$$

Therefore, for every natural *n*  >  0 it holds

(9) $$\begin{array}{c} \left[f*\left(g*h\right)\right]\left(n\right)=\left[\left(f*g\right)*h\right]\left(n\right)\text{.} \end{array}$$

And so

(10) $$\begin{array}{c} f*g=g*f\text{.} \end{array}$$

Next, consider the numerical function *ϵ* defined by the relations:

(11) $$\begin{array}{c} \epsilon \left(1\right)=1\text{,}\\ \epsilon \left(n\right)=0\text{.} \end{array}$$

For every *f* ∈ *A* and natural *n*  >  1, we have

(12) $$\begin{array}{c} \left(f*\epsilon \right)\left(n\right)=\underset{ab=n}{\sum }\;f\left(a\right)\epsilon \left(b\right)=f\left(n\right)\text{,} \end{array}$$

where *f* *∗* *ϵ*=*f*. As the operation  *∗*  is transitive, the relation *ϵ* *∗* *f*=*f* is also valid. So, the function *ϵ* is the neutral element for associative multiplication. Therefore, the pair (*A*, *∗*) is a permutable monoid. (*G*, *∗*) is a monoid and *ϵ* its neutral element. A subset H of *G* is called a submonoid of *G* if *ϵ* ∈ *H* and for every *x*, *y* ∈ *H* it holds *x∗y* ∈ *H*; that is, the pair (*H*, *∗*) is also a monoid with a neutral element *ϵ*.

Based on the above view, (*A*, *∗*), (*B*, ⋄), an d(*C*, ▹) are monoids with neutral elements eA, eB, and eC, respectively, and *f*  : *A* ⟶ *B*, *f*  : *B* ⟶ *C* are monoid morphisms. We will show that the expression *g* ◦ *f* is a monoid morphism since, for every *x*, *y* ∈ *A*, the composition of two morphisms of monoids is a monoid morphism, which is proved by the following relation [36, 38, 41]:

(13) $$\begin{array}{c} \left(g\circ f\right)\left(x*y\right)=g\left(f\left(x*y\right)\right)\\ =g\left(f\left(x\right)⋄f\left(y\right)\right)\\ =g\left(f\left(x\right)\right)▹g\left(f\left(y\right)\right)\\ =\left(g\circ f\right)\left(x\right)▹\left(g\circ f\right)\left(y\right)\text{.} \end{array}$$

Also, it holds

(14) $$\begin{array}{c} \left(g\circ f\right)\left(e_{A}\right)=g\left(f\left(e_{A}\right)\right)\\ =g\left(e_{B}\right)\\ =e_{C}\text{.} \end{array}$$

This hypothesis creates a process where the data in a machine learning system is predicted with high accuracy (any anomalies are recognized) even when they come slightly modified [22, 33]. The output of the intelligent mechanism can now be considered as a recognition of the input data's shifted prediction, based on the isomorphism of monoids that may appear in the unknown data set (assuming a uniform distribution which, although unknown, includes properties of monoid theory). That is, the output of the intelligent mechanism approaches the displaced version of the input as the intelligent system is trained. The machine learning system learns to distinguish displaced samples using this approach, resulting in highly generalized algorithmic frameworks for detecting abnormalities [19, 20].

Given that the synthesis of two monoid morphisms is a monoid morphism, proving that the inverse representation of a monoid isomorphism is likewise a monoid isomorphism suffices for implementing this mechanism [36, 37, 39].

So, considering (*M*, *∗*), (*N*, ⋄) monoids and *f*  : *M* ⟶ *N* isomorphism of monoids, if *y*_1_, *y*_2_ ∈ *N*, then there exist *x*_1_, *x*_2_ ∈ *M* with *y*_1_=*f*(*x*_1_) and *y*_2_=*f*(*x*_2_). The above formulation is related to the hypothesis of a supervised learning problem, where a set of training with N samples, {X,Y} = {*x*_*i*_, *y*_*i*_}_*i*=1_^*N*^, where xi ∈ *R*^*n*_*i*_^, *y*_i_ is a no-dimensional binary vector with only one input (corresponds to the class *x*_*i*_) equal to a multidimensional categorization process, where ni and no are the input and output dimensions, respectively. Unlabeled data helps study the data structure of the accessible data set, but classified data aids in learning. With this in mind, we have [36, 41]

(15) $$\begin{array}{c} f^{-1}\left(y_{1}⋄y_{2}\right)=f^{-1}\left(f\left(x_{1}\right)⋄f\left(x_{2}\right)\right)\\ =f^{-1}\left(f\left(x_{1}*x_{2}\right)\right)\\ =\left(f^{-1}\circ f\right)\left(x_{1}*x_{2}\right)\\ =I_{G}\left(x_{1}*x_{2}\right)\\ =x_{1}*x_{2}\\ =f^{-1}\left(y_{1}\right)*f^{-1}\left(y_{2}\right)\text{.} \end{array}$$

If eM and eN are the neutral elements of *M* and N, respectively, then *f*(*e*_*M*_)=*e*_*N*_ and therefore *f*^−1^(*e*_*N*_)=*e*_*M*_ and *f*^−1^ is a monoid morphism.

(*G*, *∗*) is a monoid with neutral elements *e* and *x* ∈ *G*. Assume that *y* ∈ *G* exists such that

(16) $$\begin{array}{c} x*y=e=y*x\text{.} \end{array}$$

In this case, the element *y* is unique because if *y*′ is another element with this property, then

(17) $$\begin{array}{c} y=y*e\\ =y*\left(x*y^{\prime }\right)\\ =\left(y*x\right)*y^{\prime }\\ =e*y^{\prime }\\ =y^{\prime }\text{.} \end{array}$$

So, the element *y* is symmetric to *x*. Also, the symmetric of *y* is *x*.

But since in a monoid each element does not always have a symmetric, then *f* must be calculated which has a symmetric element *g* (associative inverse of *f*). If and only if *g* *∗* *f*=*e*, which is equivalent to (1)*f*(1)=1, then [38, 40]

(18) $$\begin{array}{c} \underset{ab=n}{\sum }\;g\left(a\right)f\left(b\right)=0\text{,} \end{array}$$

for every natural *n*  >  1. In general, for every natural *n*  >  1, it applies

(19) $$\begin{array}{c} f^{*}\left(n\right)=-\frac{1}{f\left(1\right)}\underset{st=n,t<n}{\sum }\;f\left(s\right)f^{*}\left(t\right)\text{.} \end{array}$$

Therefore, *f* has an associative inverse if and only if *f*(1) ≠ 0. The derivative of the function is

(20) $$\begin{array}{c} f_{\Delta }\left(t\right)=\frac{du_{\Delta }\left(t\right)}{dt}=\left\{0|,|t|<|0|\;|1|/|\Delta |,|0|\leq |t|\leq |\Delta \right.\;0,t\geq \Delta \text{.} \end{array}$$

So, the data set is obtained as a subscale of the signal processing process for analyzing and manipulating the physical quantities that define the given problem of information systems security [42]. Thus, when Δ ⟶ 0, the duration of the pulse decreases and its height increases, but the area remains constant and equal to the unit. So, we study the function *f* (*t*) as an operator that acts on other functions that are smooth at points 0. Thus, we can express the function *f*(*t*) as [43–45]

(21) $$\begin{array}{c} \overset{+\infty }{\underset{-\infty }{\int }}\;f\left(t\right)\varphi \left(t\right)dt=\varphi \left(0\right)\text{,} \end{array}$$

where *φ* (*t*) is a test function, for *f*(*t*)=0 and *t* ≠ 0. So, the above process can be generalized to describe the time-shifted data expressed by the functionf (t-t_0) ADDIN ZOTERO_ITEM CSL_CITATION {″citationID″:″l8CicIUS″,″properties″:{″formattedCitation″:″ [46]\\uc0\\u8211{┤} [47]″,″plainCitation″:″ [46–48]″,″noteIndex″:0},″citationItems″:[{″id″:47,″uris″:[″http://zotero.org/users/local/knpFELzr/items/RISE.2017.8378144″,″event″:″2017 International Conference on Recent Innovations in Signal processing and Embedded Systems (RISE)″,″page″:″153–156″,″source″:″IEEE Xplore″,″title″:″Moving object detection using self adaptive Gaussian Mixture Model for real-time applications″,″author″:[{″family″:″Ali″,″given″:″Syed Tariq″},{″family″:″Goyal″,″given″:″Kalpana″},{″family″:″Singhai″,″given″:″Jyoti″}],″issued″:{″date-parts″:[[″2017″,7]]}}},{″id″:277,″uris″:[″http://zotero.org/users/local/knpFELzr/items/ICOMSSC45026.2018.8941982″,″event″:″2018 International Computers, Signals and Systems Conference (ICOMSSC)″,″page″:″378–381″,″source″:″IEEE Xplore″,″title″:″A Method of Fast Extract Signal Subspace Based on the Householder Transformation″,″author″:[{″family″:″Chang″,″given″:″Yu″},{″family″:″Wan″,″given″:″Qun″},{″family″:″Xia″,″given″:″Changxiong″},{″family″:″Wan″,″given″:″Yihe″}],″issued″:{″date-parts″:[[″2018″,9]]}}},{″id″:279,″uris″:[″http://zotero.org/users/local/knpFELzr/items × 32K complex points) image, achieving real-time performance.″,″container-title″:″2016 IEEE 13th International Conference on Signal Processing (ICSP)″,″DOI″:″10.1109/ICSP.2016.7877887″,″event″:″2016 IEEE 13th International Conference on Signal Processing (ICSP)″,″note″:″ISSN: 2164–5221″,″page″:″513–517″,″source″:″IEEE Xplore″,″title″:″Design of a flexible high-performance real-time SAR signal processing system″,″author″:[{″family″:″Jin″,″given″:″Ting″},{″family″:″Wang″,″given″:″Hongxian″},{″family″:″Liu″,″given″:″Hongwei″}],″issued″:{″date-parts″:[[″2016″,8]]}}}],″schema″:″https://github.com/citation-style-language/schema/raw/master/csl-citation.json″} [46–48]:

(22) $$\begin{array}{c} \overset{+\infty }{\underset{-\infty }{\int }}f\left(t-t_{0}\right)\varphi \left(t\right)dt=\overset{+\infty }{\underset{-\infty }{\int }}f\left(t-t_{0}\right)\varphi \left(t_{0}\right)dt\\ =\varphi \left(t_{0}\right)\overset{+\infty }{\underset{-\infty }{\int }}f\left(t-t_{0}\right)dt\\ =\varphi \left(t_{0}\right)\text{.} \end{array}$$

The above relation describes the mathematical model of the sampling process applied during the application of the semisupervised learning technique of the proposed machine learning model [49–51].

For *φ*(*t*)=1, we have [52–55]

(23) $$\begin{array}{c} \overset{+\infty }{\underset{-\infty }{\int }}f\left(t\right)dt=\overset{0^{+}}{\underset{0^{-}}{\int }}f\left(t\right)dt=1\text{.} \end{array}$$

And so

(24) $$\begin{array}{c} \overset{t_{2}}{\underset{t_{1}}{\int }}f\left(t-t_{0}\right)\varphi \left(t\right)dt=\left\{\varphi |\left(t_{0}\right)\right.,t_{1}<t_{0}<t_{2}\;0,t_{0}<t_{1},t_{0}>t_{2}\text{.} \end{array}$$

However,

(25) $$\begin{array}{c} \overset{t_{2}}{\underset{t_{1}}{\int }}f\left(\tau -t\right)f\left(t-t_{0}\right)dt=f\left(t-t_{0}\right),t_{1}<t_{0}<t_{2}\text{.} \end{array}$$

According to the logic presented by the system under consideration, the error function is defined as the integral [56]:

(26) $$\begin{array}{c} erf\left(x\right)=\frac{2}{\sqrt{\pi }}\overset{x}{\underset{0}{\int }}e^{-t^{2}}dt,x>0\text{.} \end{array}$$

Also, the complementary error function is defined as the integral [57]:

(27) $$\begin{array}{c} erfc\left(x\right)=\frac{2}{\sqrt{\pi }}\overset{\infty }{\underset{x}{\int }}e^{-t^{2}}dt,x>0\text{.} \end{array}$$

So, the error function and the complementary error function satisfy the following equation:

(28) $$\begin{array}{c} er\;f\left(x\right)+er\;fc\left(x\right)=1\text{.} \end{array}$$

The above hypotheses are proved based on the observation that

(29) $$\begin{array}{c} erf\left(x\right)+erfc\left(x\right)=\frac{2}{\sqrt{\pi }}\overset{\infty }{\underset{0}{\int }}e^{-t^{2}}dt\text{.} \end{array}$$

For the calculation of the integral [58],

(30) $$\begin{array}{c} \overset{\infty }{\underset{0}{\int }}e^{-t^{2}}dt\text{.} \end{array}$$

We consider

(31) $$\begin{array}{c} t^{2}=u\Rightarrow 2t\;dt=du\text{.} \end{array}$$

Since the integration ends are the same, we have

(32) $$\begin{array}{c} \overset{\infty }{\underset{0}{\int }}e^{-t^{2}}dt=\overset{\infty }{\underset{0}{\int }}e^{-u}u^{-1/2}\frac{du}{2}\\ =\frac{1}{2}\overset{\infty }{\underset{0}{\int }}e^{-u}u^{1/2-1}du\\ =\frac{1}{2}\Gamma \left(\frac{1}{2}\right)\\ {=}^{\left(2.9\right)}\frac{\sqrt{\pi }}{2}\text{.} \end{array}$$

This fact proves the above hypotheses about the relationships of the error functions.

Finally, a self-supervised learning methodology [17, 59, 60] is an unsupervised learning method where supervised learning work is created from unlabeled input data. Simple supervised learning usually requires a lot of labeled data. Obtaining good quality labeled data is a costly and time-consuming task, especially for a complex task such as detecting anomalies. On the other hand, unlabeled information is readily available in abundance. So, the motivation behind the self-supervised learning methodology is to learn useful representations of industrial data from an unlabeled data pool using the semisupervised process and then refine the few-tagged representations for the supervised work.

The implementation of the self-supervised learning methodology will require the reconstruction loss function, which is responsible for capturing the essential features of the context of the complete categorization process. The loss function used to train an undercomplete autoencoder is called reconstruction loss, as it is a check of how well the image has been reconstructed from the input [54, 61, 62]:

(33) $$\begin{array}{c} L_{rec}\left(x\right)=||\hat{M}⊙\left(x-F\left(\left(1-\hat{M}\right)⊙x\right)\right){||}_{2}^{2}\text{,} \end{array}$$

and the adversarial loss which models the latent data entry space of the monoid morphisms in which the following is trained:

(34) $$\begin{array}{c} L_{a\;dv}=\max_{D}\;E_{x\in X}\left[\log\;\left(D\left(x\right)\right)+\log\;\left(1-D\left(F\left(\left(1-\hat{M}\right)⊙x\right)\right)\right)\right]\text{.} \end{array}$$

Joint loss was used to implement the proposed template utilizing the combination of the above functions as follows:

(35) $$\begin{array}{c} L=\lambda_{rec}L_{rec}+\lambda_{a\;dv}L_{a\;dv}\text{.} \end{array}$$

To develop representations encapsulating the underlying standard information across various regions of the data while rejecting low-level information and noise that is a local phenomenon, we use the Contrastive Predictive Coding technique [63–66]:

(36) $$\begin{array}{c} L_{\theta^{a},\theta^{+},\left\{\theta^{-}\right\}}=-\log\frac{\exp\;\exp\left(\theta^{a}\cdot \left(\theta^{+}/k\right)\right)}{\exp\;\left(\theta^{a}\cdot \left(\theta^{+}/k\right)\right)+\underset{\theta^{-}}{\sum }\exp\;\left(\theta^{a}\cdot \left(\theta^{+}/k\right)\right)}\text{.} \end{array}$$

**Figure 1:**
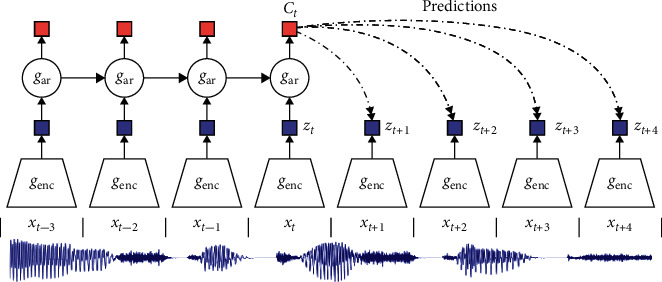
Contrastive Predictive Coding example (https://anilkeshwani.github.io/CPC/).

For example, given a lack of information, Figure 1 depicts the Contrastive Predictive Coding network, where “x” is a time series signal, data for which is available until time “t” and the model must predict the signal by the time “*t* + 4.” Here, “g_enc_” is an integration network that extracts “*z*_t_” attributes from the “*x*_t_” signal and “g_ar_” is a self-regression model that summarizes all the *z*≤ *t* in the integration space to produce a latent representation of the environment *c*_t_ = g_ar_ (*z* ≤ *t*) [67]. This composite representation is used to model a density ratio that maintains the mutual information between the predicted signal and the aggregate environment “*c*_t_” [68–71].

Thus, in the proposed system, we combine future observation predictions with a likely loss linked to whether each monoid element is always symmetric [72].

So, for a 2-class (binary) classification problem, we have [69, 71] the following.

Activation function:

(37) $$\begin{array}{c} y=\sigma \left(a\right)\equiv \frac{1}{1+\exp\;\left(-a\right)}\text{.} \end{array}$$

Probability:

(38) $$\begin{array}{c} p\left(t\;|\;x,w\right)=\overset{N}{\underset{n=1}{\prod }}\;y{\left(x_{n},w\right)}^{t_{n}}{\left\{1-y\left(x_{n},w\right)\right\}}^{1-t_{n}}\text{.} \end{array}$$

Error function:

(39) $$\begin{array}{c} E\left(w\right)=-\overset{N}{\underset{n=1}{\sum }}\left\{t_{n}\ln y_{n}+\left(1-t_{n}\right)\ln\;\left(1-y_{n}\right)\right\}\text{.} \end{array}$$

Moreover,

(40) $$\begin{array}{c} \frac{\partial E}{\partial a_{k}}=y_{k}-t_{k}\text{.} \end{array}$$

However, for classification with k-classes (multiclass), we have the following.

Activation function:

(41) $$\begin{array}{c} y_{k}\left(x,w\right)=\frac{\;\exp\;\exp\left(a_{k}\left(x,w\right)\right)}{\underset{j}{\sum }\;\exp\;\left(a_{j}\left(x,w\right)\right)}\text{.} \end{array}$$

Probability:

(42) $$\begin{array}{c} p\left(T\;|\;W\right)=\overset{N}{\underset{n=1}{\prod }}\;\overset{K}{\underset{k=1}{\prod }}\;y_{nk}^{t_{nk}}\text{.} \end{array}$$

Error function:

(43) $$\begin{array}{c} E\left(w\right)=-\overset{N}{\underset{n=1}{\sum }}\;\overset{K}{\underset{k=1}{\sum }}\;t_{kn}\ln y_{k}\left(x_{n},w\right)\text{ .} \end{array}$$

Furthermore,

(44) $$\begin{array}{c} yy_{k}=a_{k}\Rightarrow \frac{\partial E}{\partial a_{k}}=y_{k}-t_{k}\text{.} \end{array}$$

When an anomaly is run through the model, it will not recreate it since it is taught only to reproduce standard data, resulting in a considerable Mean Absolute Percentage Error (MAPE) [73, 74]:

(45) $$\begin{array}{c} \frac{100}{n}\overset{n}{\underset{i}{\sum }}\;\frac{y_{i}-{\hat{y}}_{i}}{y_{i}}\text{.} \end{array}$$

The comparison and the final categorization are achieved by defining a threshold value for MAPE, which is not sensitive to extreme values. At the same time, its values are normalized based on the actual observation, so it predicts the sample's class with high precision and recall [75].

This procedure may be repeated multiple times if it makes sense; that is, the reconstruction at each phase is adequate, implying that the new objectives are not too challenging. When a sample goes to a displaced region, it is always conceivable that it may end up in a zone with more opponents than previously. Furthermore, even if his aim puts him in a better position than before, there is a potential that the sample will be rebuilt in a worse situation. In these circumstances, repeating the operation for the troublesome pieces each time seems reasonable. That is, rediscover the problematic samples as they emerge from the categorizer's reconstruction of the details, to use the procedure again to locate the inverse of the function and to begin the process from where the previous phase of categorizing had ended. So, if the reconstruction is good enough, the procedure can detach the network from local minimums and may be done numerous times. The suggested method's most significant novelty is the simple confirmation of the results of assigning classes to an unknown collection of values using quantifiable criteria [64, 76].

Finally, we have a reduction in data dimension, clear separation of classes, and self-adaptation with this method, as the proposed system learns to reconstruct the wrong samples in the supersphere defined by computational number theory and precisely the concept of the single object to perceive the nature of an unknown state based on generalized reshaped characteristics that come directly from the unknown environment.

## 4. Conclusions

Attempting to comment on the proposed system, it is a sophisticated practice that solves an essential problem of information systems security with great accuracy and reliability. With the proposed methodology currently presented and simulated by mathematical modeling, the artificial intelligence algorithm leads to a high learning rate, which is determined by how fast the industrial system converges. In general, self-adaptation and self-learning functionality enable identifying and maintaining fundamental characteristics of complex patterns that grow and contribute to the timely and accurate forecast of circumstances completely relevant to the industrial environment.

The proposed technique significantly strengthens the methodology because, in this problem of high complexity under consideration, the results of the prediction eliminate the variability, which is attributed to the sensitivity of industrial data. This complicated connection identifies and captures the minute distinctions that set them out amid the chaotic din. The suggested technique assures that the correlation of monoid operations can be anticipated using an intelligent correlation algorithm, efficiently using multiple learning cores and matching machine learning algorithmic structures with the single-mode process.

Furthermore, an additional benefit derived from the suggested function is that it provides better prediction and a more stable categorization rate since the general behavior of the model minimizes the overall probability of an awful decision that may be associated with occurrences such as this notion. This is because modern industrial data generators generate data in huge quantities and at high speed. The result is an increase in flow data. Extracting useful information from flow data is a challenge because its nature imposes constraints that cannot be satisfied by classical learning algorithms. Stream data is infinitely large, so it is not stored in memory, and each snapshot is usually and only accessible once. So, the snapshots are not available from the beginning as they arrive at a fast pace. Also, every snapshot is processed within a short time, and access to the actual price is limited. Most important, however, is the possibility of a change in the essential data production function, which is predicted with high reliability by the proposed system.

It is critical to stress that the quality of model adaption is interpreted as a percentage of “prediction self-improvement” owing to the higher rate of categorization accuracy fluctuation using this approach. The high percentages of accuracy reached after the general convergence of the reconstructed samples represent the temporal bias induced towards the dynamics of a model at a particular moment.

Another critical interpretation that emerges from the proposed algorithm's methodology is the characteristics of the relatively low rate of “mutation” in the changes that characterize the data shift, which allows the discovery of local extremes that may be included in a learning context, given the exploration of new areas of the multidimensional solution space. On the other hand, if the rate of “mutation” was too great, it might restrict the utilization of regions of high appropriateness in the solution space and imprison the system in nongeneralizable solutions.

The basic model is high speed, owing to the limits on the connections between the hidden and visible units that make it up, as mentioned above. Because of the algorithm hidden layer's function, where the teams of one level rely solely on branches of the other level, it also efficiently and precisely detects high-level correlations in data sets. Another significant feature of the proposed method is its ability of separating and rejecting random noise in the training set. The addition of automation to reclassifying complex data as a future extension of the proposed system is essential. This is the most realistic way of operating and using intelligent systems in the operational security of modern industrial infrastructures and systems.

Suggestions for future development and enhancements to this system should also concentrate on further improving the settings of the heuristic approach of redefining and rearranging the issue samples utilized to obtain an even more efficient, accurate, and quicker classification process. Finally, it is critical to investigate the expansion of this algorithm for the analysis and classification of real-time data presented in streams so that it can completely automate identifying even stealth zero-day attack types.[77]

## Data Availability

The data can be obtained from the corresponding author upon reasonable request.
